# Parenting Styles as a Moderator of the Association between Pubertal Timing and Chinese Adolescents’ Smoking Behavior

**DOI:** 10.3390/ijerph18178903

**Published:** 2021-08-24

**Authors:** Hui Ling, Yaqin Yan, En Fu, Amin Zhu, Jianren Zhang, Siyang Yuan

**Affiliations:** 1 Psychology Department, Hunan Normal University, Changsha 410081, China; yanyaqin_hnnu@126.com (Y.Y.); aminzhu_phychology@163.com (A.Z.); jianrenzhang_hnnu@126.com (J.Z.); 2 College of Chengnan, Hunan First Normal University, Changsha 410205, China; 3 Irving Medical Center, Columbia University, New York, NY 10032, USA; En.Fu@nyspi.columbia.edu; 4 School of Dentistry, University of Dundee, Scotland DD1 4HN, UK; s.z.yuan@dundee.ac.uk

**Keywords:** puberty timing, parenting style, smoking behavior

## Abstract

Background: Pubertal timing refers to the timing of an individual regarding pubertal sexual maturation, both physiologically and psychologically. Existing research shows that pubertal timing is associated with behavioral problems. This study investigated the role of parenting style in the relationship between pubertal timing and Chinese adolescents’ smoking behavior. Methods: The study examined the association of pubertal timing, parenting style and adolescents’ smoking behavior, using the Pubertal Development Scale (Chinese version), Simplified Parenting Style Scale-Chinese version, and three items related to adolescents’ smoking situation. Participants were 1391 Chinese adolescents aged 11–16 years old (53.41% boys). Hierarchical linear regression analyses assessed the moderating role of parenting style on the association between pubertal timing and adolescent smoking behavior. Results: The results indicated that parenting style moderates the relationship between pubertal timing and adolescent smoking behavior. For male adolescents, father rejection moderated the relationship between early pubertal timing and smoking behavior. For female adolescents, father rejection, father emotional warmth, and mother emotional warmth moderated the relationship between pubertal timing and smoking behavior. Conclusions: Findings from the study highlight the importance of parenting style, which may influence the negative outcomes associated with early pubertal timing and can help improve interventions aimed at reducing these negative outcomes.

## 1. Introduction

Puberty is the transitional stage between childhood and adulthood. It is one of the most critical developmental stages and is characterized by sexual maturation, both physiologically and psychologically [1]. Pubertal timing refers to individual differences in the timing of pubertal development [2]. An individual’s pubertal timing can be categorized as early, moderate, or late, depending on when the pubertal developments occur compared to those of a reference group [3]. Precocious puberty is a condition characterized by an unusually early onset of puberty. As a general rule, precocious puberty is defined as an onset of puberty before the age of eight years in girls and before the age of nine years in boys [4]. This group will not be examined in the present study.

Pubertal timing is influenced by both genetic and environmental factors [5]. The developmental readiness hypothesis posits that entering puberty earlier than one’s peers may add psychological stress due to asynchrony among biological, social, and emotional developments [6]. Early pubertal timing is related to many externalizing behavior problems such as relational aggression, conduct problems, substance use, and smoking behaviors [7,8,9].

Contextual amplification theory provides a framework for explaining behavioral problems in adolescents. The integrated peer socialization/contextual amplification model, for example, asserts that contextual conditions that promote access to older friends and opposite-sex relationships and early pubertal timing together increase the risk of developing unhealthy and risky behavior in female adolescents [10]. Family environment, as an important contextual factor for adolescent development, can be both a risk factor and a protection factor [11]. The family environment could be a risk factor for externalizing behavior problems when adolescents with early pubertal timing received strict and inconsistent parenting. The family environment could also be a protective factor against externalizing behavior problems when parents provide a supportive and advantageous home environment to adolescents. Despite anecdotal evidence showing parenting practices as a moderator between early pubertal timing and attention deficit/hyperactivity disorder, oppositional defiance disorder, and conduct disorder [12], parenting practices interacted with early pubertal timing when predicting aggressive behavior [13,14]. Little is known regarding the role of parenting style in the relationship between pubertal timing and adolescent externalizing behavior problems. The current study focuses on a specific externalizing behavior problem in adolescents—smoking. Smoking is a direct threat to adolescent health. Turbin and colleagues compared smoking with other problems or health behaviors and found that adolescent cigarette smoking fits the problem behavior scheme rather than the health-compromising behavior scheme [15]. On average, smokers die ten years earlier than nonsmokers and over 80 percent of smokers started smoking in adolescence or childhood [16].

Existing research indicates that early pubertal timing and parenting style might influence smoking behavior in youth. For example, disengaged parenting practices are related to more significant risks of smoking initiation [17]. Students who are current smokers perceived their parents as less authoritative and more permissive than students who were not smokers [18]. However, parenting style does not influence all parameters of smoking [2,17]. Therefore, the primary goal of the current study is to investigate the relationships among parenting style, pubertal timing, and adolescent smoking behaviors. 

Evidence suggests that cultural norms might influence the relationship between parenting style and behavior problems in youth. Usually, less authoritative and more permissive parenting style is related to a higher risk for adolescents to start smoking. However, when the parenting practice is acceptable with the youth’s cultural norm, there are not necessarily deleterious consequences in their children’s development [19,20]. Mexican–American and Euro–American families were examined regarding the relationship between mother acceptance, hostile control, and children’s conduct problems and depressive symptoms. Results revealed a strong relationship between mother acceptance and conduct problems in Spanish-speaking Mexican–American families but not in the English-speaking Mexican–American families. Further examination showed that mother acceptance and hostile control were unrelated in English-speaking Mexican–Americans, positively related for Spanish-speaking Mexican–Americans, and negatively related in Euro–Americans. By collecting data from a predominantly collectivist culture, such as Chinese [21], the current study hopes to contribute to the understanding of cultural influence on parenting and behavioral problems in youth. This is the secondary objective of the current study.

Existing studies on the relationship between pubertal timing and adolescent smoking behavior found different results in girls and boys. Girls who mature early had a higher rate of regular smoking behavior, younger age upon first smoking incidence, and more frequent smoking behavior than girls who mature on average timing or relatively late [22]. However, male adolescents lack consistent results regarding the relationship between early pubertal timing and smoking behavior as revealed in existing research. Early research suggests that early pubertal timing benefits boys because peers view them as calm, well-composed, and mature [23]. Late maturity is a risk factor for psychosocial difficulties and mental health problems, such as adaptation problems. Recent research, however, asserts that early pubertal timing in boys correlates with a higher risk of smoking, higher smoking rate, and higher smoking frequency [2,22]. Therefore, the third purpose of the current study is to investigate the different mechanisms through which parenting style influences the relationship between early pubertal timing and smoking behavior in male and female adolescents. 

The current study aimed to investigate the relationships among pubertal timing, parenting style, and smoking behavior in male and female Chinese adolescents. We hypothesized that the relationships between pubertal timing and smoking behavior depends on parenting style. We also expected that the mechanisms through which parenting style moderate the relationships between pubertal timing and smoking behavior differ between male and female adolescents.

## 2. Materials and Methods

### 2.1. Participants 

Participants were 1391 seventh to ninth graders recruited from eight middle schools in the Hunan province of China. Cluster sampling methods were performed to choose classes from each school, totaling 1540 students as potential participants. Complete data were available for 1391 students (743 boys and 648 girls), resulting in a 90.32% response rate. 

### 2.2. Measures

#### 2.2.1. Demographic Information

Demographic information was collected through a set of questions at the beginning of the questionnaire: gender, age, grade, school, residential address, father’s education level and occupation, mother’s education level and occupation, number of siblings, and parental marital status.

#### 2.2.2. Pubertal Timing 

Pubertal timing was measured by the Pubertal Development Scale (PDS). This scale was initially developed by Peterson [24] and is used to evaluate physiological development in late childhood and early adolescence. In the current questionnaire, the Pubertal Development Scale includes a male version and a female version. Each version includes three common items and two gender-dependent items. The three common items are sudden increase in height, body hair growth, and skin change. In the male version, two items were added in addition to the three common items: voice change and facial hair growth. In the female version, two items were added in addition to the three common items: breast development and period. There are two levels of values for period: “0” indicates “no period” and “1” indicates “has period”. The rest of the items have 4 levels of values: “1” indicates “have not started”, “2” indicates “just started”, “3” indicates “started”, and “4” indicates “finished”. Therefore, higher PDS total score indicates earlier pubertal timing among peers. This scale has good validity and reliability, Cronbach’s α ranged from 0.68 to 0.83. Higher PDS total score indicates earlier pubertal timing among peers. Chan and colleagues [25] translated and revised the Pubertal Development Scale based on Chinese adolescent population. Their translated scale indicates a Kendall τ–b index between self-report PDS and evaluator PDS as 0.61 in females and 0.49 in males. This study was its reliance upon self-report assessments of pubertal development, which did not use the clinical standards.

#### 2.2.3. Parenting Style 

Parenting style was measured using the Simplified Parenting Style Scale (Chinese version) [26]. Psychometric characteristics of this scale were obtained from 708 Chinese undergraduate students (age 17–25, 525 females). The Parenting Style Scale used in the current study includes 21 father items and 21 mother items, measuring three dimensions of parenting practices reported by the youth: rejection, emotional warmth, and over protection. Each item was measured on a 4-point Likert scale (1 = “never”, 2 = “occasional”, 3 = “often”, 4 = “always”). This questionnaire has relatively good validity and reliability, Cronbach’s α ranged from 0.74 to 0.84 on all dimensions. Test–retest reliability ranged from 0.70 to 0.81.

#### 2.2.4. Smoking Behavior 

Smoking behavior was measured using three items. The first item was “which of the following option best describes your smoking behavior?” (1) never smoked (2) smoked 1 or several times for fun (3) used to smoke on regular basis but have been stopped for more than 3 months (4) smoke occasionally, about 1 cigarette every week (5) smoke regularly, 1–6 cigarette every week (6) smoke regularly, more than 7 cigarettes every week. Responses were categorized into “never smoked” (response 1), “tried smoke” (2 and 3), and “currently smoking” (4, 5, and 6). This categorization method conformed to the WHO definition for minors’ smoking behavior and was shown to be effective in revealing adolescences smoking situation in existing study [20]. The second and third items measured participants’ frequency of smoking within one month prior to the study and during their lifetime. A 6-point Likert scale was used in these two items: from “never” to “more than 40 times”. “1” means “never”, “6” means “more than 40 times”. The Cronbach’s α for this scale is 0.86.

### 2.3. Procedure

Before questionnaire administration, we obtained approval from the Ethical Committee for Scientific Research in the Hunan Normal University. All participants were fully informed of the purpose and characteristics of the study and signed the informed consent. With the help of class teachers, trained research assistants explained issues on questionnaire completion following standardized instruction in class. Questionnaires were completed in class and collected on-site. To ensure confidentiality, all questionnaires were completed anonymously. Participants were informed that they could choose not to respond to the questionnaire or any question on the questionnaire if they felt uncomfortable with responding.

### 2.4. Data Analysis

All analyses were conducted in SPSS version 22.0 (SPSS, Inc., Chicago, IL, USA). One participant was excluded from the analyses because she chose “no period” but filled in the age of menarche. Three abnormal menarche age participants were excluded from the analyses (0, 2, 8). The number of respondents who reported “tried smoking” were combined with the number of respondents who self-identified as “current smoker” to calculate the numbers for “smoking rate”. Three values of pubertal timing (relative to same-age peers) were calculated for each gender. Twelve groups were identified based on gender and age. Each individual PDS score was standardized within his or her group. Individuals with a standardized score over 1 were categorized as “early pubertal timing”; individuals with standardized score less than –1 were categorized as “late pubertal timing”; the rest of the participants were categorized as “average pubertal timing” [25,27]. Table 1 presents PDS categories in groups of genders and ages.

Descriptive statistics for study sample were presented first. Second, Chi–square tests were used to investigate individual differences in main variables. Third, Pearson correlations were used to estimate the bivariate correlations among the variables. Finally, hierarchical linear regression analyses were conducted to examine the hypothesis that parenting style moderates the relationship between pubertal timing and adolescent smoking behavior; both pubertal timing scores and smoking scores were centralized (subtract the mean from the value) before testing possible moderation effect [28]. This study has used grade and hometown as control variables.

Furthermore, considering that all variables were reported by the adolescents, we used Harman’s single-factor test to assess the common method variance [29]. If the common method bias was serious, only a single factor would emerge to account for most of the covariance in all variables. We performed a factor analysis on all items and found that 17 factors with eigenvalues greater than one were extracted, with Factor 1 accounting for 15.82% of the variance (less than 40%), which suggested that common method variance is not of substantial concern in the present study.

## 3. Results

Table 2 presents the frequency (%) of the characteristics of adolescents by adolescents’ smoking status and results of association (*N* = 1391), as well as pubertal timing differences on smoking groups. The final sample consisted of 1391 respondents aged 11 to 16. During the month preceding the study, the smoking rate among boys was 21.53%. Among these smokers, 17.50% tried smoking and 4.04% are current smokers. Moreover, there was gender difference (χ^2^ = 11.20, *p* < 0.01), grade difference (χ^2^ = 18.94, *p* < 0.01), hometown difference (χ^2^ = 8.15, *p* < 0.05), and pubertal timing differences (χ^2^ boys = 10.68, *p* < 0.05; χ^2^ girls = 8.78, *p* = 0.067) in the Chinese adolescents’ smoking behaviors (Table 2).

In addition, bivariate correlations among primary study variables are reported in Table 3. As shown, PDS, father rejection, father over-protection, mother rejection, and mother over-protection were significantly and positively related to boys’ smoking behaviors. Father emotional warmth and mother emotional warmth were significantly and negatively related to boys’ smoking behaviors. Moreover, PDS, father rejection, and mother rejection were significantly and positively related to girls’ smoking behaviors. Father emotional warmth and mother emotional warmth was significantly and negatively related to girls’ smoking behaviors. Father over-protection and mother over-protection were not significantly related to girls’ smoking behaviors.

In boy respondents, hierarchical linear regression analyses were conducted to evaluate the moderating effects of adolescents’ parenting style (measured by centralized dimension scores) between pubertal timing (measured by centralized PDS total scores) and boys’ smoking behavior (measured by scores on the first smoking question). As shown in Table 4, the main effect of father rejection was statistically significant. A significant two-way father rejection × the total score of pubertal development scale interaction was found. That is, father rejection significantly moderated the relation between PDS and boys’ smoking behaviors.

**Figure 1:**
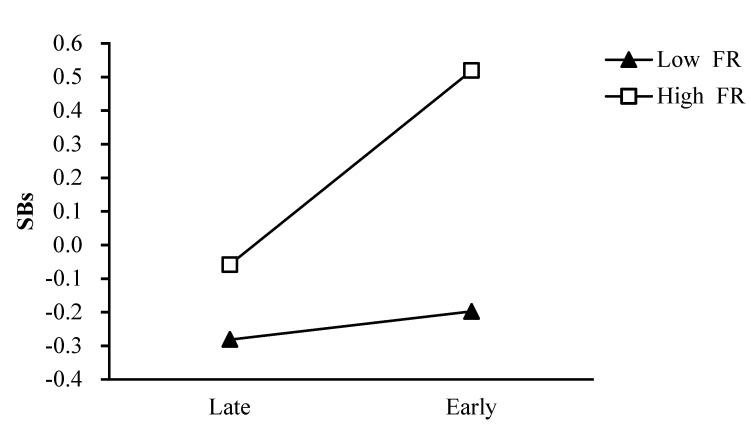
The two−way interaction between PT and FR in predicting boys’ SBs. Note: PT pubertal timing, FR father rejection, SBs smoking behaviors.

Regarding the moderating role of father rejection, simple slope analyses indicated that father rejection had an intensifying effect on the relation between pubertal timing and boys’ smoking behaviors. In the present study, values at 1SD above and below the mean of father rejection were used to estimate the simple slopes of the association between pubertal timing and adolescents’ smoking behaviors. The effect of pubertal timing on boys’ smoking behaviors was greater for boys with a high level of father rejection (simple slope = 0.29, *t* = 5.82, *p* < 0.001) than for boys with a low level of father rejection (simple slope = 0.04, *t* = 0.84, *p* = 0.40); Figure 1.

In girl respondents, hierarchical linear regression analyses were conducted to evaluate the moderating effects of parenting style between pubertal timing and girls’ smoking behavior. As shown in Table 4, the main effect of father rejection was statistically significant. A significant two-way father rejection × the total score of pubertal development scale interaction was found. That is, father rejection significantly moderated the relation between PDS and girls’ smoking behaviors. The main effect of father emotional warmth was statistically significant. A significant two-way father emotional warmth × the total score of pubertal development scale interaction was found. That is, father emotional warmth significantly moderated the relation between PDS and girls’ smoking behaviors. Moreover, the main effect of mother emotional warmth was statistically significant. A significant two-way mother emotional warmth × the total score of pubertal development scale interaction was found. That is, mother emotional warmth significantly moderated the relation between PDS and girls’ smoking behaviors. 

**Figure 2:**
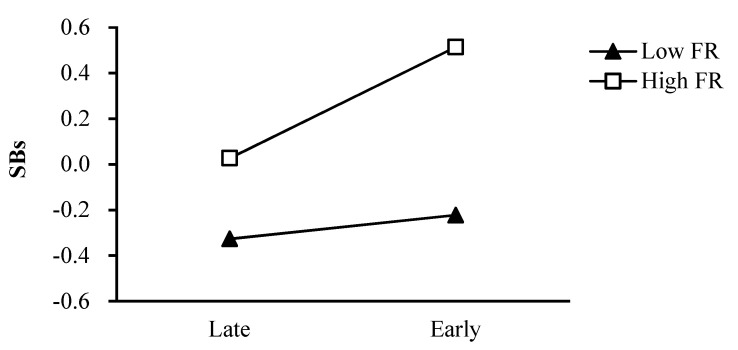
The two−way interaction between PT and FR in predicting girls’ SBs. Note: PT pubertal timing, FR father rejection, SBs smoking behaviors.

**Figure 3:**
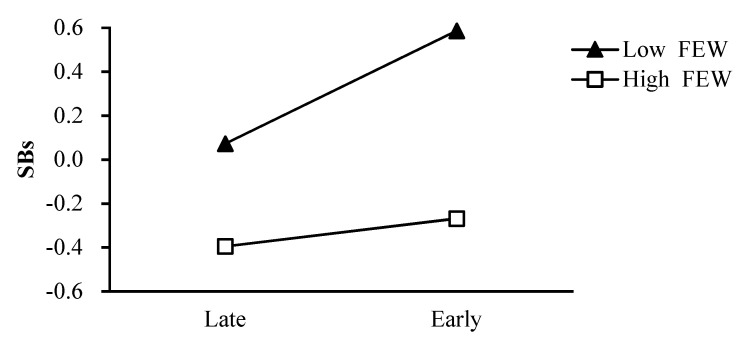
The two−way interaction between PT and FEW in predicting girls’ SBs. Note: PT pubertal timing, FEW father emotional warmth, SBs smoking behaviors.

**Figure 4:**
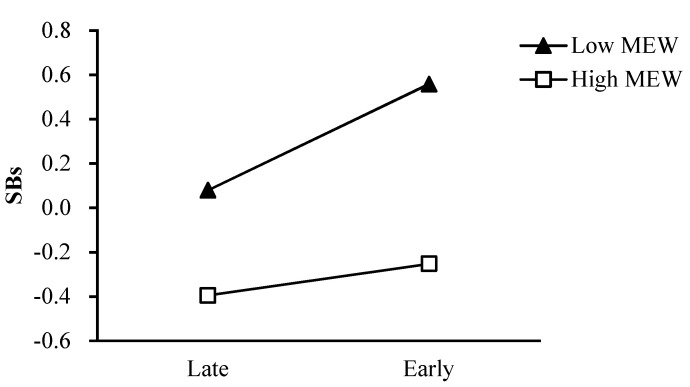
The two−way interaction between PT and MEW in predicting girls’ SBs. Note: PT pubertal timing, MEW mother emotional warmth, SBs smoking behaviors.

As shown in Figure 2, the effect of pubertal timing on girls’ smoking behaviors was greater for girls with a high level of father rejection (simple slope = 0.24, *t* = 4.93, *p* < 0.001) than for girls with a low level of father rejection (simple slope = 0.05, *t* = 1.00, *p* = 0.32). Regarding the moderating role of father emotional warmth in the relation between pubertal timing and girls’ smoking behaviors, the effect of pubertal timing on girls’ smoking behaviors was greater for girls with a low level of father emotional warmth (simple slope = 0.26, *t* = 4.93, *p* < 0.001) than for girls with a high level of father emotional warmth (simple slope = 0.06, *t* = 1.26, *p* = 0.21; Figure 3). Regarding the moderating role of mother emotional warmth in the relation between pubertal timing and girls’ smoking behaviors, the effect of pubertal timing on girls’ smoking behaviors was greater for girls with a low level of mother emotional warmth (simple slope = 0.24, *t* = 4.49, *p* < 0.001) than for girls with a high level of mother emotional warmth (simple slope =0.07, *t* = 1.40, *p* = 0.16; Figure 4).

## 4. Discussion

The current study investigates the relationship between pubertal timing and adolescent smoking behaviors and the moderating effect of parenting style on this relationship. Pubertal timing has both biological and social implications. Early maturing individuals could have difficulty adjusting to their change physically and socially and therefore have further behavioral problems [13]. Previous evidence has indicated that earlier pubertal timing was associated with a higher likelihood of smoking behavior. This may in part be that while adolescent’s self-consciousness and self-esteem are increasing, they begin to have their own independent interests and opinions; they are hoping to be treated as adults, thus adolescents would do things against the will of parents and teachers, such as smoking and drinking. The gender-related role expectations theory indicates that in early adolescence, adolescents will have an accelerated socialization process of gender differences, that is, girls are more likely to adopt female stereotypes and likewise boys are more likely to adopt male stereotypes [30]. Earlier pubertal timing may accelerate the socialization of gender, and smoking is one of the male stereotypes [31]. There is a cross-cultural consistency in the relationship between adolescents’ smoking behavior and pubertal timing [7,8,9,21].

We found the mechanisms through which parental style moderates the relationship between pubertal timing and adolescent smoking behavior differed in males and females. In both male and female adolescents, father rejection moderated the relationship between pubertal timing and smoking behavior. In male adolescents, mother rejection moderated the relationship between pubertal timing and smoking behavior. In female adolescents, father emotional warmth and mother emotional warmth moderated the relationship between pubertal timing and smoking behavior. 

In male adolescents, paternal rejection moderated the relationship between pubertal timing and smoking behavior. Results from simple slope testing show that high level of paternal rejection increase smoking behavior in male adolescent with early pubertal timing. High paternal emotional warmth, on the other hand, could compensate for the risk that early pubertal timing has on male adolescents’ smoking behavior. Adolescents in the puberty period are fragile and experience sensitive feelings. A high level of paternal rejection might become a source of negative stimulus and induce feelings, such as upset or defeat in adolescents, thus increase the negative effects of early pubertal timing on adolescents’ smoking tendency. In the relationship with parents, emotional warmth from father has special meaning for male adolescents. While interacting with their fathers, male adolescents learn gender role behaviors from modeling and behavior enforcement patterns in their fathers. In fact, fathers have larger impact on adolescents than mothers regarding anxiety, self-esteem, depressive symptoms, and puberty development [32,33]. 

In female adolescents, paternal rejection, paternal emotional warmth, and maternal emotional warmth moderated the relationship between pubertal timing and smoking behavior. Results from simple slope testing shows that low levels of maternal emotional warmth would increase female adolescents’ smoking behavior regardless of their pubertal timing status. One reason for this result might be that many Chinese families honor the “strict father and loving mother” parenting style. Mothers are expected to provide children with warmth, care, and love [34]. Therefore, children who experience low levels of emotional warmth from their mothers are not likely to experience enough care and love. The current study also found that high levels of paternal rejection would increase smoking behavior in female adolescents with early pubertal timing. Paternal emotional warmth, on the other hand, could compensate for the negative impact of early pubertal timing on female adolescents. One possible reason is that high levels of paternal rejection would lead to a sense of defeat in adolescents. Furthermore, adolescents view their fathers’ behaviors as role models and would treat others in school with similar interactions [35]. Therefore, negative father parenting style, such as high parental rejection and low parental emotional warmth would lead to maladaptive peer relationships in adolescents and increase negative impacts of early pubertal timing in adolescents [36]. Moreover, females often gain a sense of security and protection through interaction with their fathers [15]. Emotional warmth that girls gain from their fathers might have protected them from the negative influence of early pubertal timing.

Several of our results also concurred with those in the existing literature. First, the current study shows that adolescents with early pubertal timing reported a higher smoking rate than their peers with average or late pubertal timing [2,9,21]. Second, smoking behaviors in adolescents are associated with parenting style [37]. 

Some potential limitations have also been identified in this study. First, all measures were based on a self-reported questionnaire completed by adolescents in a public setting. This could potentially compose a biased sample because different data had been obtained from student-report of parenting style or parent-report of parenting style [18]. Future studies could collect data from both adolescents and their parents. Second, due to the limitations of human, financial, and material resources, this study adopts just one measure of pubertal timing, i.e., PDS. PDS has been widely used when judging the pubertal status of adolescents. It is usually used to compare the PDS scores of adolescents with the same gender peer of the same age, with +/− 1 SD of z-sore as the criterion [25,27]. Although self-assessment of PDS is probably sufficiently accurate for large-scale studies wanting to assess maturational changes [38], it could be improved by having a nurse or physician to assess Tanner Stages in future [39]. Third, fewer than five female participants self-identified as “current smoker” in each grade, rendering validity threats for many statistical tests. Future studies could adopt a different categorization system to better suit the data. Fourth, this sample came from 11–16 year-old Chinese adolescents. Special caution should be paid when translating the findings into a different research setting given the differences in the age range and culture. Thus, the conclusions drawn may not readily generalize to youth with different age composition or adolescents of other cultural backgrounds. The current study focuses on a collectivism culture (Chinese culture) [21]. Collectivism implies that life satisfaction of an individual depends on the individual’s fulfillments of his or her obligations in social roles [40]. Collectivism also values restraint in emotional expression as a means of ensuring in-group harmony. In Chinese families, strict or authoritarian parenting strategies are often considered as a means of maintaining organizational control to facilitate family harmony [41]. It is a cultural norm for Chinese parents to display relatively low levels of emotional intimacy and high levels of over-protection when being compared with parenting styles in European families, Latino families, and other Asian families. Therefore, it is possible that certain measures of parenting style among our participants displayed a ceiling effect or floor effect, thus influenced the testing of the moderator role of parenting style on the relationship between pubertal timing and adolescent smoking behaviors. Future studies could investigate the relationships between pubertal timing, parenting style, and adolescent smoking behaviors in different cultures. Last, since adolescence is a complex age of life, there might be other factors that interplay with smoking behavior. This should be taken into consideration when interpreting the current findings. Future longitudinal research should explicitly examine between and within individual changes.

## 5. Conclusions

The current study indicated that early pubertal timing could increase smoking rate in middle school students and that parenting style moderated the relationship between pubertal timing and adolescent smoking behavior. It is suggested that paternal emotional warmth and maternal emotional warmth are essential sources of support for adolescents who are stressed. Emotional connection with parents and other significant people can help release the negative impact of adverse situations [42]. Future studies are needed to clarify other variables (e.g., peer relationship, socioeconomic status, autonomy) that may have an interplay with adolescents’ smoking behavior.

## Figures and Tables

**Table 1:** PDS categories in groups of genders and ages.

Gender	Age	Early *N* (%)	Average *N* (%)	Late *N* (%)
Boys	11 (*n* = 8)	0 (0.0)	6 (75.0)	2 (25.0)
	12 (*n* = 134)	24 (17.9)	91 (67.9)	19 (14.2)
	13 (*n* = 180)	27 (15.0)	129 (71.7)	24 (13.3)
	14 (*n* = 166)	38 (22.9)	98 (59.0)	30 (18.1)
	15 (*n* = 217)	44 (20.3)	133 (61.8)	40 (18.4)
	16 (*n* = 38)	3 (7.9)	27 (71.1)	8 (21.1)
Girls	11 (*n* = 16)	4 (25.0)	9 (56.3)	3 (18.8)
	12 (*n* = 161)	34 (21.1)	101 (62.7)	26 (16.1)
	13 (*n* = 140)	31 (22.1)	92 (65.7)	17 (12.1)
	14 (*n* = 186)	34 (18.3)	134 (72.0)	18 (9.7)
	15 (*n* = 129)	21 (16.3)	84 (65.1)	24 (18.6)
	16 (*n* = 16)	4 (25.0)	9 (56.3)	3 (18.8)

**Table 2:** Frequency (%) of the characteristics of adolescents by adolescents’ smoking status and results of association (*N* = 1391), pubertal timing differences on smoking groups.

		SR *N* (%)	NS *N* (%)	TS *N* (%)	CS *N* (%)	χ^2^	*p*
Boys/Girls	Boys (*n* = 743)	160 (21.53)	583 (78.47)	130 (17.50)	30 (4.04)	11.20	0.004
	Girls (*n* = 648)	98 (15.12)	550 (84.88)	86 (13.27)	12 (1.85)		
Grade	Seven (*n* = 341)	43 (12.61)	298 (87.39)	32 (9.38)	11 (3.23)	18.94	0.001
	Eight (*n* = 486)	85 (17.49)	401 (82.51)	75 (15.43)	10 (2.06)		
	Nine (*n* = 564)	130 (23.05)	434 (76.95)	109 (19.33)	21 (3.72)		
Hometown	City (*n* = 500)	73 (14.60)	427 (85.40)	62 (12.40)	11 (2.20)	8.15	0.017
	Rural (*n* = 891)	185 (20.76)	706 (79.24)	154 (17.28)	31 (3.48)		
Boys	Early (*n* = 136)	41 (30.15)	95 (69.85)	30 (22.06)	11 (8.09)	10.68	0.03
	Average (*n* = 484)	97 (20.04)	387 (79.96)	82 (16.94)	15 (3.10)		
	Late (*n* = 123)	22 (17.89)	101 (82.11)	18 (14.63)	4 (3.25)		
Girls	Early (*n* = 128)	29 (22.66)	99 (77.34)	25 (19.53)	4 (3.13)	8.78	0.067
	Average (*n* = 429)	53 (12.35)	376 (87.65)	47 (10.96)	6 (1.40)		
	Late (*n* = 91)	16 (17.58)	75 (82.42)	14 (15.39)	2 (2.20)		

SR smoking rate, NS never smoked, TS tried smoking, CS current smoker.

**Table 3:** Correlations among primary study variables.

		PDS	Smoking Behaviors
Boys	PDS	-	0.17 ***
	Father rejection	0.04	0.23 ***
	Father emotional warmth	−0.01	−0.14 ***
	Father over-protection	0.01	0.10 *
	Mother rejection	0.04	0.20 ***
	Mother emotional warmth	−0.02	−0.06 *
	Mother over-protection	0.02	0.10 **
Girls	PDS	-	0.16 ***
	Father rejection	0.02	0.29 ***
	Father emotional warmth	−0.01	−0.33 ***
	Father over-protection	0.05	0.04
	Mother rejection	0.12 **	0.19 ***
	Mother emotional warmth	−0.03	−0.32 ***
	Mother over-protection	0.11 **	0.04

PDS the total scores of pubertal development scale. * *p* < 0.05 ** *p* < 0.01 *** *p* < 0.001.

**Table 4:** Regression analyses testing parenting style as moderators of the relation between pubertal timing and smoking behavior.

		Variable	*β*	*R* ^2^	*R*^2^ Change	*F*
Girls	Step 1	PDS	0.15 ***	0.11	0.11	39.94 ***
		FR	0.27 ***			
	Step 2	PDS × FR	0.10 **	0.12	0.12	7.55 **
	Step 1	PDS	0.16 ***	0.14	0.14	51.31 ***
		FEW	−0.33 ***			
	Step 2	PDS × FEW	−0.10 **	0.15	0.14	36.94 **
	Step 1	PDS	0.16 ***	0.13	0.13	47.36 ***
		MEW	−0.32 ***			
	Step 2	PDS × MEW	−0.08 *	0.14	0.13	33.52 *
Boys	Step 1	PDS	0.17 ***	0.09	0.08	34.17 ***
		FR	0.24 ***			
	Step 2	PDS × FR	0.12 ***	0.10	0.10	27.13 ***

PDS the total scores of pubertal development scale, FR Father rejection, FEW Father emotional warmth, MEW Mother emotional warmth. * *p* < 0.05, ** *p* < 0.01, *** *p* < 0.001.

## Data Availability

The data presented in this study are available on request from the corresponding author. The data are not publicly available due to protect teenagers’ privacy.
